# 3.P. Round table: The Commercial Determinants of Health: Industry tactics shaping science, policy and our environment

**DOI:** 10.1093/eurpub/ckac129.197

**Published:** 2022-10-25

**Authors:** Organised by: LSHTM (UK), Chair persons: Mark Petticrew (UK)

## Abstract

Prevailing narratives on health and illness emphasise individual choice and product ‘misuse’ for the disease and environmental burdens associated with harmful industries, deflecting from the wider environment in which these choices are made. Public Health frameworks such as the Dahlgren and Whitehead model enable us to consider the broader socio-economic, cultural and environmental conditions that influence these choices (or lack thereof). Until recently, the influence of multinational corporations on these broader conditions has often been ignored, despite the global impact these corporations have in shaping science, policy and our environment. Increasingly Public Health academics and advocates are calling for an enhanced focus on these ‘commercial determinants of health’ (CDoH) to understand the ways through which corporate interests affect health, sustainability, and health inequities. The concept of CDoH considers (among other things) the harmful or damaging nature of certain products and the tactics employed by industries to protect their commercial interests. The harmful tactics employed by the tobacco industry are well documented, but there is growing evidence that similar tactics are employed by other harmful industries more generally, from alcohol, gambling and food, to agrichemical and fossil fuel industries. In addition to driving consumption (with resulting population and planetary ill health), these tactics have a cross-industry, complementary, cumulative effect; spreading misinformation, shaping ‘personal responsibility’ and ‘nanny state’ narratives, weakening regulation and undermining science and policymaking. The impact of these harms accumulates across the life-course, with differential exposure to both harmful products and tactics driving health inequities. We argue there is a need to make visible, or ‘resurface’ these tactics; at present, Public Health often fails to challenge corporate strategies and, more concerningly, can be complicit in reinforcing industry framings of issues. This session will outline how a life-course, complex systems approach can support this endeavour, exploring a range of industry strategies through a series of case studies and discussing actions needed to safeguard public understanding and policy from commercial interests. This workshop will include 3 short presentations to illuminate some of the more insidious and less overt tactics, with opportunities for questions and panel discussion.

The CDoH is an emerging field in Public Health and this session will give audience members an introduction to industry strategies and influences within a life-course framework, focusing on less overt channels of influence and some of the lesser-known industries.

**Key messages:**

• The commercial determinants of health present a significant threat to public and planetary health but are conspicuously absent in the majority of public health conceptual frameworks.

• Framing these hidden harms and exposures within the life-course model can support awareness of CDoH and facilitate public health professionals to counter corporate influences.

**Speakers/Panellists:**

**Claire Mulrenan**

LSHTM, London, UK

**May Van Schalkwyk**

LSHTM, London, UK

**Nason Maani**

LSHTM, London, UK

